# Clinical Utility of Postoperative Day 1 Technetium‐99m Mercaptoacetyltriglycine Scintigraphy for Early Assessment of Graft Function in Living Donor Kidney Transplant Recipients

**DOI:** 10.1155/joot/4619384

**Published:** 2026-05-25

**Authors:** Shun Nakazawa, Yoshitaka Sekine, Yusuke Tsuji, Takanori Shimizu, Mai Onose, Yuta Maeno, Azusa Kanayama, Tatsuhiro Sawada, Akira Ohtsu, Yoshiyuki Miyazawa, Yuji Fujizuka, Seiji Arai, Masashi Nomura, Hidekazu Koike, Hiroshi Matsui, Motoaki Hatori, Toshiyuki Tanaka, Kazuhiro Suzuki

**Affiliations:** ^1^ Department of Urology, Gunma University Hospital, Maebashi, 371-8511, Gunma, Japan, gunma-u.ac.jp; ^2^ Department of Urology, Hidaka Hospital, Takasaki, 370-0803, Gunma, Japan; ^3^ Department of Urology, Public Tomioka General Hospital, Tomioka, 370-2393, Gunma, Japan

**Keywords:** eGFR, graft function, living donor kidney transplantation, MAG3 clearance, renal scintigraphy

## Abstract

**Background:**

At our institution, 99mTc‐MAG3 renal scintigraphy is routinely performed preoperatively in living kidney donors and on postoperative Day 1 in recipients. Given the interindividual variability in MAG3 clearance, we hypothesized that postoperative MAG3 clearance could serve as an early indicator of renal graft function. In addition, we explored whether the donor‐to‐recipient clearance ratio (M‐ratio) provides supplemental clinical value. Early identification of such indicators of graft function is essential for optimizing postoperative management and improving outcomes.

**Methods:**

This retrospective study analyzed 52 living donor kidney transplants performed between October 2009 and May 2024. Associations were examined between donor and recipient MAG3 clearance values, the M‐ratio (elevated ≥ 1.5 vs decreased/stable < 1.5), donor/recipient age and sex, T1/2 pattern (good excretion ≤ 20 min vs delayed > 20 min or unmeasurable), total ischemic time, graft weight, dialysis duration, and estimated glomerular filtration rate (eGFR) at 1 week and 1–12 months postoperatively. Variables with *p* < 0.05 in univariate analysis entered multivariate regression.

**Results:**

Univariate analysis showed that recipient MAG3 clearance, the M‐ratio, donor and recipient age, T1/2 (up to 1 month), and graft weight (at 1 week) were significantly associated with graft function. In contrast, donor MAG3 clearance was not correlated with postoperative renal function. In multivariate analysis, recipient MAG3 clearance, recipient age, and graft weight remained independently associated with early graft function, whereas the M‐ratio lost significance.

**Conclusions:**

Recipient MAG3 clearance on postoperative Day 1 is a practical, noninvasive indicator of renal graft function, while donor clearance and the M‐ratio are not independently associated with postoperative outcomes.

## 1. Introduction

Renal dynamic scintigraphy is a valuable imaging modality for assessing renal impairment, evaluating urinary tract obstruction, and evaluating residual renal function before planned nephrectomy [1]. Among the radiopharmaceuticals used for dynamic renal imaging, technetium‐99m mercaptoacetyltriglycine (99mTc‐MAG3) and technetium‐99m diethylenetriamine pentaacetic acid (99mTc‐DTPA) are most commonly employed. 99mTc‐MAG3 is actively secreted by the proximal renal tubules and reflects effective renal plasma flow [2], whereas 99mTc‐DTPA is filtered by the glomeruli and excreted into the urine without tubular reabsorption, thereby serving as an indicator of glomerular filtration rate [1]. Several studies have reported that 99mTc‐MAG3 has faster clearance and provides superior image quality compared with 99mTc‐DTPA, making it particularly useful for evaluating transplanted kidneys and patients with impaired renal function [3]. MAG3 clearance is calculated from renal uptake measured within the first 1‐2 min after radiotracer administration [4], and it is widely regarded as a reliable quantitative measure of renal function [5].

At our institution, 99mTc‐MAG3 scintigraphy is routinely performed as part of the evaluation process for living donor kidney transplantation: preoperatively in donors to assess split renal function and guide kidney selection and on postoperative Day 1 in recipients to evaluate renal perfusion and tubular function. MAG3 clearance values are known to vary considerably among individuals, both in donors and recipients. However, no studies have yet examined the relationship between MAG3 clearance values and postoperative graft function. Given that MAG3 clearance is a reliable measure of renal function, we hypothesized that both the absolute values and the donor–recipient ratios could serve as indicators of postoperative graft function, prompting us to undertake this study. Because living donor kidneys generally demonstrate favorable baseline quality and minimal ischemic injury, early quantitative markers capable of detecting subtle functional differences may be particularly valuable in this population.

## 2. Methods

This retrospective observational study aimed to identify potential early indicators of renal graft function following living donor kidney transplantation by evaluating the associations between 99mTc‐MAG3 clearance, both absolute values and the donor‐to‐recipient ratio, and various clinical parameters.

We analyzed 52 consecutive living donor kidney transplants performed at our institution between October 2009 and May 2024. Postoperative graft function was assessed using the estimated glomerular filtration rate (eGFR), measured at five time points: 1 week, 1 month, 3 months, 6 months, and 12 months after transplantation.

MAG3 clearance was determined using 99mTc‐MAG3 renal scintigraphy. The donor‐to‐recipient clearance ratio (M‐ratio) was calculated by dividing the recipient’s MAG3 clearance by that of the donor. Patients were categorized into two groups according to the M‐ratio: elevated (≥ 1.5) and decreased/stable (< 1.5). The cutoff value of 1.5 was selected based on exploratory analysis, which identified it as the threshold yielding the greatest separation in postoperative eGFR between groups. Renogram curves were also used to determine the time required for radionuclide counts to decrease by half (T1/2), with T1/2 ≤ 20 min classified as good excretion and T1/2 > 20 min or unmeasurable excretion classified as delayed.

Variables assessed for association with postoperative eGFR included the M‐ratio, absolute MAG3 clearance values in both donors and recipients, donor and recipient age and sex, T1/2 classification, graft weight, total ischemic time (TIT), and pretransplant dialysis duration. At our institution, intraoperative renal biopsy is routinely performed after reperfusion and ureteral anastomosis, prior to wound closure, in all living donor kidney transplant recipients. Histopathological evaluation demonstrated that most grafts were classified as Banff Category 1, indicating relatively homogeneous baseline histological findings in this cohort. Statistical analyses were performed using EZR Version 1.61. Differences in mean eGFR between two groups (e.g., by sex, T1/2 category, or M‐ratio category) were evaluated using Student’s *t*‐test; Welch’s correction was applied when variances were unequal, and the Wilcoxon rank‐sum test was used when normality was questionable. Continuous variables were assessed using Pearson’s product‐moment correlation coefficient. Variables with a *p* value < 0.05 in univariate analysis were entered into multivariate regression models to identify independent indicators of graft function at each postoperative time point. Statistical significance was set at *p* < 0.05.

## 3. Results

### 3.1. Patient Characteristics

Baseline characteristics of the study population are summarized in Table 1. A total of 52 living donor kidney transplants were analyzed. Among the donors, 22 (42%) were male and 30 (58%) were female, with a mean age of 62.0 ± 9.9 years. The recipient cohort comprised 27 males (52%) and 25 females (48%), with a mean age of 41.0 ± 14.7 years. Donor MAG3 clearance demonstrated relatively low variability, with a mean of 157.4 ± 41.0 mL/min, whereas recipient MAG3 clearance showed a wider distribution, with a mean of 195.1 ± 159.5 mL/min. Based on the M‐ratio, 19 recipients (37%) were classified into the elevated group (M‐ratio ≥ 1.5) and 33 (63%) into the decreased/stable group (M‐ratio < 1.5). T1/2 values were evenly distributed, with 26 recipients (50%) in the good excretion group (T1/2 ≤ 20 min) and 26 (50%) in the delayed excretion group (T1/2 > 20 min or unmeasurable). The mean TIT was 114.0 ± 32.6 min, and the mean graft weight was 180.0 ± 46.2 g. Including 20 preemptive transplants (without prior dialysis), the mean duration of dialysis before transplantation was 1.0 ± 5.1 years.

**TABLE 1:** Patient background and postoperative renal function.

*Variable*	

Age (D)/years old	62 ± 9.9
Age (R)/years old	41 ± 14.7
Sex (D)	Male 22
	Female 30
Sex (R)	Male 27
	Female 25
MAG3 clearance (D) mL/min	157.4 ± 41.0
MAG3 clearance (R) mL/min	195.1 ± 159.5
M‐ratio	Rising group 19
	Decreasing/stable group 33
T1/2	Good excretion group 26
	Delayed excretion group 26
TIT/min	114 ± 32.6
Graft weight/g	180 ± 46.2
Dialysis period/year	1 ± 5.1

*Postoperative renal function*	*Mean ± SD*

eGFR 1 week after surgery mL/min/1.73 m^2^	46.9 ± 24.4
eGFR 1 month after surgery mL/min/1.73 m^2^	43.7 ± 12.2
eGFR 3 months after surgery mL/min/1.73 m^2^	39.9 ± 13.4
eGFR 6 months after surgery mL/min/1.73 m^2^	39.1 ± 12.6
eGFR 12 months after surgery mL/min/1.73 m^2^	40.1 ± 13.9

*Note:* Values are expressed as number or mean ± SD.

Abbreviations: D, donor; R, recipient; SD, standard deviation; TIT, total ischemic time.

### 3.2. Postoperative Renal Function

Post‐transplant renal function, assessed by eGFR, is summarized in Table 1. At 1 week post‐transplantation, the mean eGFR was 46.9 ± 24.4 mL/min/1.73 m^2^. The mean eGFR was 43.7 ± 12.2 mL/min/1.73 m^2^ at 1 month, 39.9 ± 13.4 mL/min/1.73 m^2^ at 3 months, 39.1 ± 12.6 mL/min/1.73 m^2^ at 6 months, and 40.1 ± 13.9 mL/min/1.73 m^2^ at 12 months, demonstrating relatively stable renal function during the chronic phase.

### 3.3. Univariate Analysis

**FIGURE 1:**
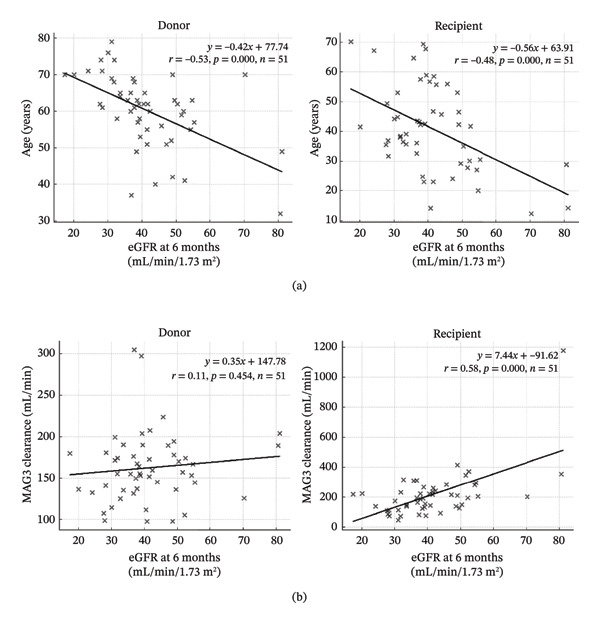
Post‐transplant renal function vs. age and recipient MAG3 clearance. (a) Relationship between 6‐month eGFR and donor/recipient age. (b) Relationship between 6‐month eGFR and recipient 99mTc‐MAG3 clearance measured on postoperative day.

The associations between clinical and demographic factors, including donor and recipient age, sex, MAG3 clearance values, the M‐ratio, T1/2, TIT, graft weight, dialysis duration, and postoperative renal function, were assessed using univariate analysis, with the results summarized in Table 2. Both donor and recipient age showed significant negative correlations with eGFR at all postoperative time points, with younger age in both groups consistently associated with better graft function (Figure 1). Recipients who received kidneys from male donors tended to have higher eGFR values than those receiving kidneys from female donors although this difference did not reach statistical significance. Recipient sex was not significantly associated with postoperative eGFR. Recipient MAG3 clearance was positively correlated with eGFR at all time points (Figure 1), indicating that higher clearance values were associated with improved graft function. Conversely, donor MAG3 clearance showed no significant correlation with eGFR at any time point.

**TABLE 2:** Indicators and postoperative renal function (univariate analysis).

eGFR	1 W	1 M	3 M	6 M	12 M
Age (D)					
*p* value	0.0136	< 0.01	< 0.01	< 0.01	< 0.01
CC	−0.34	−0.52	−0.437	−0.534	−0.411
Age (R)					
*p* value	< 0.01	< 0.01	< 0.01	< 0.01	< 0.01
CC	−0.409	−0.386	−0.408	−0.484	−0.411
Sex (D)					
*p* value	0.067	0.092	0.177	0.094	0.386
Sex (R)					
*p* value	0.513	0.609	0.652	0.627	0.784
MAG3 clearance (D)					
*p* value	0.74	0.604	0.256	0.454	0.403
CC	0.047	0.073	0.162	0.107	0.123
MAG3 clearance (R)					
*p* value	< 0.01	< 0.01	< 0.01	< 0.01	< 0.01
CC	0.658	0.504	0.626	0.584	0.682
M‐ratio					
*p* value	< 0.01	< 0.01	0.017	< 0.01	0.029
T1/2					
*p* value	< 0.01	0.024	0.42	0.213	0.305
TIT					
*p* value	0.676	0.971	0.431	0.477	0.902
CC	0.059	−0.005	0.113	0.102	−0.018
Graft weight					
*p* value	< 0.01	0.084	0.087	0.147	0.335
CC	0.37	0.249	0.25	0.213	0.147
Dialysis period					
*p* value	0.201	0.982	0.855	0.681	0.674
CC	−0.18	0.003	−0.026	0.059	0.062

Abbreviations: CC, correlation coefficient; D, donor; M, months after surgery; R, recipient; TIT, total ischemic time; W, a week after surgery.

Patients in the elevated M‐ratio group (≥ 1.5) had significantly higher eGFR than those in the decreased/stable group (< 1.5) throughout the 12‐month follow‐up. T1/2 classification was significantly associated with eGFR at 1 week and 1 month, with patients in the good excretion group (T1/2 ≤ 20 min) demonstrating higher values. However, this association was not observed beyond 3 months postoperatively. Graft weight showed a weak but statistically significant positive correlation with eGFR at 1 week, but the association was not present at later time points. TIT and pretransplant dialysis duration were not significantly correlated with postoperative eGFR at any time point.

### 3.4. Multivariate Analysis

Multivariate linear regression analyses were conducted to identify independent indicators of postoperative renal graft function using variables that demonstrated statistically significant associations with eGFR in univariate analysis. Candidate variables included donor age, recipient age, recipient MAG3 clearance, the M‐ratio, T1/2 classification, and graft weight. Donor age, recipient age, recipient MAG3 clearance, and the M‐ratio were included in the regression models for all postoperative time points (1 week, 1 month, 3 months, 6 months, and 12 months). T1/2 classification was included for the 1‐week and 1‐month models, and graft weight was included for the 1‐week model only. Results are summarized in Table 3. In multivariate analysis, donor age was significantly associated with graft function at 1, 3, and 6 months post‐transplantation, whereas recipient age showed significant negative associations with eGFR at 1, 3, 6, and 12 months. Recipient MAG3 clearance was an independent indicator of eGFR at 1 week and at 3, 6, and 12 months postoperatively. Conversely, the M‐ratio was not significantly associated with graft function at any time point. Similarly, T1/2 classification showed no significant associations with eGFR at 1 week or 1 month. Graft weight was independently associated with better graft function only at 1 week after transplantation.

**TABLE 3:** Indicators and postoperative renal function (multivariate analysis).

eGFR	1 W	1 M	3 M	6 M	12 M
Age (D)					
*p* value	0.072	< 0.01	0.039	< 0.01	0.078
Age (R)					
*p* value	< 0.01	0.038	0.032	< 0.01	0.032
MAG3 clearance (R)					
*p* value	0.01	0.176	< 0.01	< 0.01	< 0.01
M ratio					
*p* value	0.24	0.167	0.864	0.575	0.568
T1/2					
*p* value	0.106	0.131			
Graft weight					
*p* value	0.018				

Abbreviations: D, donor; M, months after surgery; R, recipient; W, a week after surgery.

## 4. Discussion

At our institution, 99mTc‐MAG3 scintigraphy is routinely performed preoperatively in living kidney donors and on postoperative Day 1 in recipients undergoing living donor kidney transplantation. In most cases, the left kidney is selected for procurement to preserve renal vein length; however, if preoperative scintigraphy reveals a difference in split renal function exceeding 10%, the right kidney may be chosen. In recipients, postoperative MAG3 scintigraphy is used to assess renal perfusion, detect anastomotic stenosis, and evaluate for ureteral obstruction. Although the use of donor 99mTc‐MAG3 scintigraphy to evaluate split renal function is well established [6], some transplant centers have discontinued the practice due to concerns about cost and radiation exposure [7]. Conversely, recipient MAG3 scintigraphy remains a key tool for postoperative assessment of allograft function and early detection of complications and is considered superior to other imaging modalities [8]. Given our institution’s routine use of MAG3 scintigraphy in both donors and recipients, we investigated whether additional insights, particularly MAG3 clearance values and their ratios, could be derived from these studies. Our findings demonstrated that recipient MAG3 clearance was significantly associated with postoperative graft function across all time points in both univariate and multivariate analyses, whereas donor MAG3 clearance showed no association. Although the M‐ratio was significant in univariate analysis, it did not retain predictive value in multivariate models, likely due to its strong correlation with recipient MAG3 clearance. These results suggest that recipient‐specific factors, particularly early postoperative clearance, exert a greater influence on graft outcomes than the relative donor–recipient clearance ratio. The cutoff value for the M‐ratio in this study was set at 1.5, determined empirically from exploratory analysis of our cohort as the threshold producing the greatest separation in postoperative eGFR between groups. As no prior studies have established an optimal threshold, this value should be regarded as provisional and validated in larger cohorts.

Previous studies have highlighted the prognostic value of MAG3 scintigraphy parameters in transplant recipients. For instance, the Hilson perfusion index has been shown to correlate with graft function for up to 12 months post‐transplant [9]. Similarly, delayed excretion and reduced function patterns observed on postoperative renograms have been linked to lower graft function and poorer long‐term outcomes [10]. However, these investigations primarily focused on qualitative imaging patterns rather than absolute MAG3 clearance values. To our knowledge, the present study is the first to demonstrate a quantitative association between recipient MAG3 clearance and postoperative graft function, as well as to evaluate the predictive value of the M‐ratio. Notably, although intraoperative biopsies indicated that most grafts had minimal baseline pathological changes (Banff Category 1), we observed significant interindividual variability in recipient MAG3 clearance. This suggests that POD‐1 MAG3 scintigraphy may detect subtle early functional differences that are not fully reflected by routine histological assessment.

Regarding renogram excretion patterns, our study found that T1/2 classification was associated with eGFR only in the early postoperative period (up to 1 month) and not thereafter. This limited association may be explained by the stronger predictive value of recipient age and MAG3 clearance, which could overshadow the influence of renogram pattern alone. Furthermore, in our study, scintigraphy was performed on postoperative Day 1, whereas previous studies assessed renogram patterns on days 3 or 7, when perfusion and function may be more stable.

Donor and recipient age were both inversely correlated with graft function, consistent with previous reports [11]. In terms of graft weight, our findings align with a prior study showing that graft weight—and, more precisely, the cortical volume‐to‐recipient weight ratio—predicted renal function at 1‐month post‐transplantation [12]. That study further demonstrated that the cortical volume‐to‐recipient weight ratio was a more accurate predictor than graft weight alone. Our results also support the potential value of computed tomography volumetry over nuclear scintigraphy in donor evaluation, particularly given that donor MAG3 clearance did not correlate with graft outcomes [13].

This study has several limitations. First, the relatively small sample size may limit both its statistical power and generalizability. Second, residual confounding cannot be excluded. Although we adjusted for major donor‐ and surgery‐related factors such as donor age, TIT, and graft weight, other intraoperative variables, including vascular anatomical complexity (e.g., multiple renal arteries) and intraoperative hemodynamic fluctuations, were not specifically analyzed and may have influenced postoperative graft function. Third, follow‐up was restricted to the first postoperative year, a period during which graft function is predominantly influenced by surgical and donor factors. Conversely, longer‐term outcomes are shaped by additional factors such as rejection and infection. Fourth, the accuracy of MAG3 clearance measurements may be influenced by patient body habitus, particularly in obese individuals, due to photon attenuation and depth‐related signal loss. As we did not routinely collect or adjust for detailed anthropometric data such as body mass index, this potential source of systematic bias remains a limitation of this retrospective analysis. Finally, from a conceptual standpoint, POD‐1 MAG3 clearance should be interpreted as an early indicator of graft function rather than a strict predictor, as both MAG3 clearance and subsequent eGFR reflect renal function through related physiological pathways. Nevertheless, POD‐1 MAG3 clearance provides an objective and quantitative assessment of graft function at a very early postoperative stage, when serum creatinine may not yet be stable, supporting its clinical utility. Future studies with larger cohorts and extended follow‐up are needed to validate these findings and to identify indicators of long‐term graft survival and immune‐mediated events.

## 5. Conclusion

This study demonstrated that recipient MAG3 clearance measured on postoperative Day 1 is a significant independent early indicator of short‐term graft function following living donor kidney transplantation. Conversely, donor MAG3 clearance and the M‐ratio were not reliable indicators of postoperative renal function. These results suggest that early postoperative MAG3 scintigraphy in recipients can provide valuable quantitative information for assessing graft outcomes, whereas routine MAG3 evaluation in donors may have limited clinical utility. Further prospective studies are needed to validate these findings and clarify their relevance to long‐term graft prognosis.

## Author Contributions

Shun Nakazawa designed the study and drafted the manuscript. Yoshitaka Sekine contributed to the clinical management of patients, data interpretation, and critically revised the manuscript. Yusuke Tsuji, Takanori Shimizu, Mai Onose, Yuta Maeno, Azusa Kanayama, Tatsuhiro Sawada, Akira Ohtsu, Yoshiyuki Miyazawa, Yuji Fujizuka, Seiji Arai, Masashi Nomura, Hidekazu Koike, Hiroshi Matsui, Motoaki Hatori, and Toshiyuki Tanaka contributed to patient recruitment, data collection, and clinical management. Kazuhiro Suzuki supervised the entire project.

## Funding

This research did not receive any specific grant from funding agencies in the public, commercial, or not‐for‐profit sectors.

## Ethics Statement

This study was approved by the Institutional Review Board of Gunma University Hospital (approval No.: 1337) and was conducted in accordance with the Declaration of Helsinki and the Declaration of Istanbul. Due to the retrospective nature of the study, the requirement for written informed consent was waived, and an opt‐out approach was adopted.

## Consent

Please see the Ethic Statement.

## Conflicts of Interest

The authors declare no conflicts of interest.

## Data Availability

The data that support the findings of this study are available on request from the corresponding author. The data are not publicly available due to privacy or ethical restrictions.
